# Testing the role of the FcγRIIB immunoreceptor tyrosine-based inhibitory motif in regulation of the B cell immune response

**DOI:** 10.1002/iid3.64

**Published:** 2015-06-04

**Authors:** Raja Vuyyuru, Shixue Shen, Tim Manser

**Affiliations:** Department of Microbiology and Immunology, Thomas Jefferson UniversityPhiladelphia, PA, USA

**Keywords:** antibody response, B cell development, Fc receptors, inhibitory motif, transgenic mice

## Abstract

In vitro studies have demonstrated that the immunoreceptor tyrosine-based inhibitory motif (ITIM) of the inhibitory Fc receptor FcγRIIB is critical for mediating attenuation of signaling via immunoreceptor tyrosine-based activation motif (ITAM) containing receptors, such as the B cell antigen receptor (BCR), when FcγRIIB is co-cross-linked to these activation receptors. To test the role of the FcγRIIB ITIM motif in regulation of the B cell immune response in vivo, we constructed lines of transgenic mice expressing a form of FcγRIIB with an inactivating tyrosine (Y) to phenylalanine (F) mutation in the ITIM motif. Detailed studies of one of these lines, in which the mutant FcγRIIB was expressed on B cells and other cell types that normally express this receptor, were performed. No quantitative differences in germinal center (GC) B cell responses were observed between the mutant FcγRIIB transgenic line and control mice. However, serum antibody and antibody forming cell responses were often observed to be elevated in the ITIM mutant FcγRIIB transgenic mice as compared to controls, though not to the same extent as mice deficient in expression of FcγRIIB. Moreover, primary B cells from the ITIM mutant FcγRIIB line did not display the same level of augmented BCR signaling as primary FcγRIIB deficient B cells under conditions inducing co-cross-linking of FcγRIIB and the BCR. In total, these data suggest that a functional ITIM motif is not required for all in vivo inhibitory activity of this receptor. However, we also found that the transgenic ITIM mutant FcγRIIB receptor was expressed at abnormal levels in several hematopoietic lineages. Thus, confirmation of our findings will require the generation and analysis of mice in which an ITIM mutant form of FcγRIIB is expressed in vivo as is the endogenous receptor.

## Introduction

The IgG Fc receptor FcγRIIB is an inhibitory receptor that regulates the activity of B cells and a variety of other cell types in mice and humans 1–5. FcγRIIB is a low affinity IgG Fc receptor and so only binds IgGs in the form of immune complexes (IC). FcγRIIB contains an immuno-receptor tyrosine based inhibitory motif (ITIM) in its cytoplasmic tail 6. Alternative splicing of transcripts from the single FcγRIIB gene in mice results in two isoforms – FcγRIIB1 and FcγRIIB2 7,8. The B1 isoform contains an additional cytoplasmic domain that inhibits internalization of ICs 9. Murine B cells exclusively express FcγRIIB1 4.

The tyrosine (Y307) in the FcγRIIB ITIM is phosphorylated by Src kinases when FcγRIIB is cross-linked to ITAM-containing receptor complexes such as the B cell antigen receptor (BCR). Cross-linking of the BCR and FcγRIIB will take place on B cells in vivo when they interact with ICs containing antigen for which their BCRs are specific. The phospho-ITIM recruits the inositol polyphosphate phosphatase SHIP and, to a lesser extent the protein tyrosine phosphatases SHP-1 and SHP-2, via the SH2 domains of these proteins resulting in their activation 5,10. SHIP phosphatase activity reduces recruitment of the plextrin homology domain proteins Btk and PLCγ to the plasma membrane, and causes other downstream effects, resulting in the attenuation of B cell activation and dependent processes 11. Recruitment of SHP-1 and 2 results in dephosphorylation of activating tyrosine kinases. FcγRIIB signaling also inhibits formation of the immunological synapse on B cells 12,13, and inhibits cooperative signaling of the BCR with the BAFFR (BR3) 14 and TLR7 and 9 15. In addition, co-cross-linking of FcγRIIB and the BCR results in inhibition of the activation of Akt by BCR signaling, resulting in reduced B cell survival and increased cell death 16. We and others have shown that follicular dendritic cells (FDCs) and B cells in germinal centers (GCs) express high levels of FcγRIIB 17–21. Collectively, these findings motivated models for the GC response proposing a central regulatory role for FcγRIIB 22–24.

Mutation of the ITIM Y307 to phenylalanine (F) results in inability of FcγRIIB to recruit SHIP, SHP-1 and 2 and attenuates inhibition of BCR signaling in B cell lines in vitro. The Y307→F mutant form of FcγRIIB was found to induce cell cycle arrest and apoptosis in the DT40 chicken B cell line when extensively homologously cross-linked 25. This apoptotic response appears to require phosphorylation of FcγRIIB by ABL family kinases on a non-ITIM Y in the cytoplasmic tail 26. Both splenic and long lived bone marrow (BM) antibody forming cells (AFCs) express surface levels of FcγRIIB 2-3 fold higher than resting B cells 27. In addition, FcγRIIB mutants that lack the cytoplasmic tail are still capable of inhibiting steps in the formation of the immunological synapse on B cells 13. Many studies, including our own have shown that IC-mediated feedback of AFC activity is a central function of FcγRIIB in mice in viv*o* 4,27–31. As cross-linking of FcγRIIB on AFCs in vitro increases their rate of apoptosis 30, this suggests that the FcγRIIB apoptotic pathway is involved in regulation of the AFC response in vivo.

A fundamental role of FcγRIIB in the regulation of autoimmunity has been demonstrated in mice, where deficiencies or altered expression of this FcR contribute to auto-antibody production and development of systemic pathology 4,32–34. In humans, a variety of FcγRIIB gene polymorphisms resulting in altered expression levels or membrane distribution of this receptor are associated with development of systemic lupus erythematosus (SLE) 35–40 and rheumatoid arthritis (RA) 38,41,42. Additional, data indicate association of these alleles with a variety of other autoimmune diseases 38. It has also been proposed that FcγRIIB plays a key regulatory role in the response to many infectious pathogens in humans 38, a notion supported by studies on the responses of FcγRIIB deficient mice to model antigens 17,22,29.

Given the central role played by FcγRIIB in the regulation of the B cell response and antibody-mediated autoimmunity, and the fact that multiple activities have been attributed to this receptor, we sought to determine to what extent a functional ITIM motif in FcγRIIB was required for the actions of this receptor in vivo. To this end, we generated ITIM Y307→F mutant FcγRIIB1 transgenic mice on a C57BL/6 (B6) background. Analysis of one of these lines revealed that inhibition of in vitro BCR signaling by the mutant FcγRIIB was reduced and in vivo AFC responses were amplified as compared to wild type B cells and mice, but not to the extent displayed by FcγRIIB deficient B cells and mice. These results support the idea that ITIM-independent inhibitory activities of FcγRIIB play a role in the regulation of B cell responses in vivo.

## Materials and Methods

### Ethics statement

This study was carried out in strict accordance with the recommendations in the Guide for the Care and Use of Laboratory Animals of the National Institutes of Health. All experimental protocols involving mice were approved by the Institutional Animal Care and Use Committee of Thomas Jefferson University (generation of transgenic mice: 773D and 773M; breeding of mice: 344J; all other experimental procedures: 344I).

### Mice

C57BL/6 (B6) mice were obtained from the Jackson Laboratories and FcγRIIB deficient mice created using a B6 ES cell line (B6.FcγRIIB deficient) were a kind gift of Dr. Jeffrey Ravetch (Rockefeller University). Both of these lines were bred in house. The FY77^+/−^ and FY16^+/−^ lines, expressing a form of B6 mouse FcγRIIB1 with an Y307→F mutation in the ITIM motif were generated as follows. A construct containing a cDNA encoding this form of FcγRIIB and a splicing/poly adenylation site from the mouse β-globin gene was a kind gift from Dr. Silvia Bolland (NIAID, NIH). In this construct, the expression of the cDNA is driven by the mouse H2K promoter and the mouse IgH intronic enhancer. The insert in this construct was isolated from the plasmid backbone by restriction endonuclease digestion and gel purification and used to generate transgenic mice via injection into fertilized B6 eggs. Resulting transgenic founder mice and first generation offspring of matings to B6 mice that were found to express total levels of FcγRIIB (transgenic and endogenous) on B cells in the peripheral blood approximately twofold greater than B6 mice were then bred to B6.FcγRIIB deficient mice until the endogenous FcγRIIB gene was lost. This resulted in the FY77^+/−^ line of mice. To create the FY16^+/−^ line, the same approach was used except with a construct derived from that used to create the FY77^+/−^ line in which the H2K promoter region was replaced by standard recombinant DNA methods with a 1.1 kb fragment containing the transcriptional promoter from the mouse Vk10A antibody light chain gene.

### Immunizations

YF16^+/−^, B6.FcγRIIB deficient and B6 mice were immunized i.p. with 100 μg of 4-hydroxy-3-nitrophenyl acetyl chicken γ-globulin (NP22-CGG, Biosearch Technologies, Petaluma, CA) in alum. For primary immune responses, mice were sacrificed on day 8, and for long-lived AFC responses mice were sacrificed 30 days post immunization. Secondary immune responses were induced by i.p. boosting 4 weeks after primary immunizations with 50 μg of NP-CGG in PBS. One week later mice were sacrificed. For germinal center responses mice were immunized i.p. with 100 μL of 10% sheep red blood cells (SRBCs) (weight/volume, Lampire Biological Laboratories, Ottsville, PA) in PBS and sacrificed 9 days later. Cells and sera were harvested from all mice at the time of sacrifice.

### Antibodies and other reagents

Antibodies and other reagents used for flow cytometry and immunohistology included: FITC-peanut lectin (agglutinin) (Vector Laboratories); anti-B220 (RA3-6B2); anti-CD3∊ (145-2C11); anti-CD23 (B3B4); anti-CD21/35 (7G6); Ly-6G and Ly-6C (Gr-1) (RB6-8C5); anti-FcγRIIB/RIII (2.4G2); rat IgG Ab to mouse FDCs (FDC-M1); NK-1.1 (PK136); all purchased from BD Pharmingen, San Jose, CA; goat anti-rat IgG-biotin (Jackson Immunoresearch Laboratories, West Grove, PA); anti-CD138 (281.2); anti-IgD (11-26); anti-CD11b (M1/70); from BioLegend, CA; anti-IgM (II/41); anti-F4/80 (BM8); anti-CD11c (N418); streptavidin (SA)-APC; all from eBioscience, San Diego, CA. The K9.361 hybridoma, that expresses a mAb specific for the B6 allelic form of FcγRIIB was a kind gift from Dr. Ullrich Hammerling (Sloan-Kettering Memorial Hospital, New York, NY), and the mAb it produces was purified and biotinylated in our laboratory.

### Immunohistology

Spleens were snap frozen in OCT compound and cryosections (5–6 microns) were prepared as described 43. Immunohistology was performed using the Abs listed above. For detecting FDCs a three-step approach was used. Sections were first stained with rat anti-mouse FDC (FDC-M1), then a biotinylated anti rat IgG was used as secondary Ab, and a SA-APC conjugate was used to detect biotin. Stained sections were analyzed using a Leica DM5000B fluorescent microscope (Leica microsystems, Germany) and captured digital images were processed using the Leica Application Suite (LAS) imaging software.

### Flow cytometry

Four and eight color flow cytometric analysis was done on single cell suspensions prepared from the spleen, bone marrow and peritoneal exudate of naive and immunized mice stained with multiple combinations of the Abs listed above. Biotinylated Abs were detected with streptavidin-APC. Stained cells were analyzed using an LSRII flow cytometer (BD Biosciences, San Jose, CA). Data were analyzed using the FlowJo software (Treestar, Ashland, OR).

### ELISpot assays for primary, memory, and long lived AFCs

Multi screen 96-well filtration plates (Millipore, Billerica, MA) were coated with 4-hydroxy-3-nitrophenyl acetyl conjugated to bovine serum albumin (NP19-BSA, Biosearch Technologies, Petaluma, CA) at a concentration of 10 μg/ml overnight at 4°C. Splenocyte suspensions from immunized mice were initially plated at 1 × 10^6^ cells per well and then diluted serially (1:2) in RPMI1640 medium containing 10% FBS and incubated for six hours at 37°C. Bound antibodies were detected using biotinylated anti-mouse IgM and IgG Abs (Jackson Immunoresearch Laboratories, West, Grove, PA) followed by streptavidin conjugated to alkaline phosphatase (Vector Laboratories, Burlingame, CA). The plates were developed using the Vector Blue Alkaline–Phosphatase Substrate kit III (Vector Laboratories, Burlingame, CA). ELISpots were counted using a computerized imaging video system (Cellular Technology, Shaker Heights, OH).

## ELISA

Anti-NP specific serum Abs were measured in sera from NP-CGG immunized mice by solid-phase ELISA on 96-well plates (Immulon-4; Thermo Electron, Waltham, MA) as previously described 44.

### Calcium flux measurements

Total splenocytes were isolated from 8–10 week old YF16^+/−^, B6.FcγRIIB deficient and B6 mice and resting B cells were enriched using negative selection with anti-CD43 (Ly-48) immunomagnetic beads (MACS) according to the manufacturer's instructions (Miltenyi Biotec, Auburn, CA). The purity of isolated B cells was evaluated by flow cytometry and was always >90%. MACS purified B cells were resuspended at 2 × 10^6^/ml in HBSS with 3 μM fura-2/AM (Molecular Probes, Grand Island, NY) and were placed in a water bath at 37°C for 15 min. The cells were then washed twice and diluted to 10^6^ cells/ml in HBSS/1% FCS. Intracellular Ca^2+^ levels were then analyzed in a quartz cuvette using a fluorescence spectrometer (LS55; Perkin Elmer Life Sciences, Waltham, MA) and excitation at 340 and 380 nm. After the baseline Ca^2+^ level was established (∼50 sec) either 10 μg/ml (Fab) anti-IgM-biotin alone, or this reagent and 2.4G2-biotin antibodies were added. Then, after 50 sec cross-linking of surface IgM or co-cross-linking of FcγRIIB and IgM receptors was induced by adding 200 ng/ml streptavidin. Intracellular Ca^2+^ concentrations were calculated using a fluorescence spectrometer measurement program as described 45.

### Apoptosis assay

Two approaches were used for evaluating apoptosis after FcγRIIB receptor cross-linking on B cells in vitro. The first was done in 96 well flat bottom plates coated overnight with mAb 2.4G2 Ab at 10 μg/ml in PBS. MACS purified (anti-CD43 negative selection) splenic B cells were then plated at 1 × 10^5^ per well in 100 μl complete RPMI medium. Alternatively, MACS purified splenic B cells were cultured at the above concentration in complete RPMI medium in 96 well flat bottom plates and soluble biotin-2.4G2 added to 10 μg/ml. Streptavidin was then added at a concentration of 2.5 μg per well to cross-link the bound 2.4G2 antibodies. Levels of apoptosis were measured 24 and 48 h later in both cases by using the Annexin-V kit (BD Biosciences, San Jose, CA) and flow cytometry. In some experiments recombinant BLyS was added to the cultures at 10 ng/ml to increase general B cell viability.

### Anti-nuclear antigen antibody assay

Mice were allowed to age and bled at 18, 32, and 54 week time points. Sera were diluted 1/50 in PBS and used to stain Hep-2 cells fixed on microscope slides (Antinuclear Antibody kit, Antibodies Incorporated, Davis, CA) for 1 h at room temperature. Slides were washed in PBS and bound autoantibodies detected using an anti-mouse kappa light chain monoclonal antibody conjugated to FITC. The slides were then analyzed by fluorescence microscopy and images captured using identical magnifications and exposure times.

### Statistical analyses

Statistical significance was determined with the Prism 5 program (GraphPad Software Inc., CA) using a two-tailed, unpaired Student *t*-test.

## RESULTS

### Generation and characterization of FcγRIIB ITIM Y→F transgenic mice

**Figure 1:**
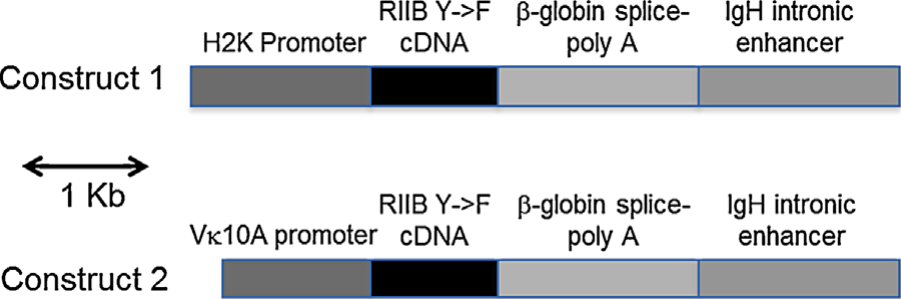
Constructs used to generate transgenic mice. The structure of the linear fragments of DNA used to generate the FY77^+/−^ and FY16^+/−^ transgenic lines of mice are illustrated schematically.

Two constructs were used to generate transgenic mice expressing the ITIM Y 307→F mutant form of FcγRIIB – one in which expression of an FcγRIIB1 Y307→F cDNA is driven by the mouse MHC class I gene H2K promoter and the mouse IgH intronic enhancer; and another in which this expression is driven by a mouse Ig Vk promoter and the mouse IgH intronic enhancer (Fig. 1). Offspring of C57BL/6 (B6) strain background founder mice were screened for levels of FcγRIIB expression on B cells in peripheral blood by flow cytometry, and one hemizygous line obtained from each construct expressing approximately twofold higher levels of FcγRIIB than on B6 B cells was then crossed to the B6.FcγRIIB knock out background. The resulting lines will heretofore be referred to as the YF77^+/−^ and YF16^+/−^ lines. The YF77^+/−^ line contains the H2K promoter/IgH enhancer construct and the YF16^+/−^ line contains the Vk promoter/IgH enhancer construct.

**Figure 2:**
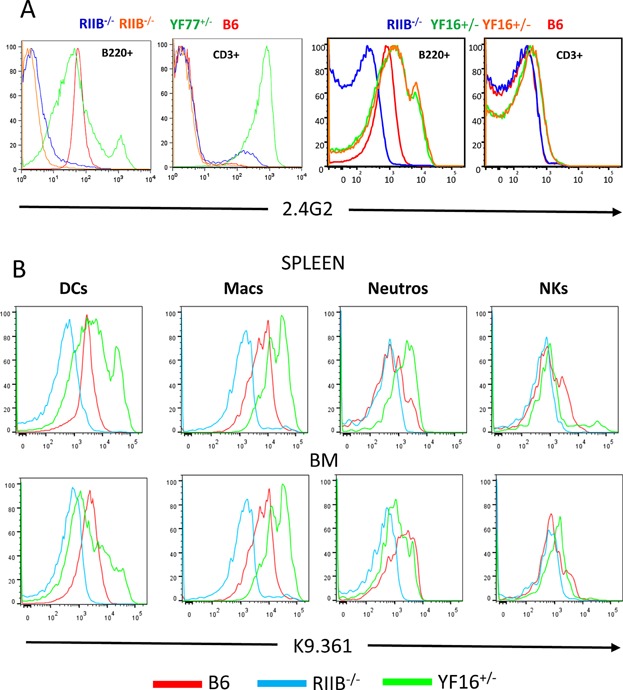
Surface levels of expression of the transgene-encoded Y307→F FcγRIIB on various cell types. Levels of 2.4G2 (anti-FcγRIIB/RIII) or K9.361 (anti-B6 FcγRIIB) staining were determined on: Panel A; Splenic B cells (B220^+^) and T cells (CD3^+^); Panel B; dendritic cells (CD11b/11c^+^), macrophages (F4/80^+^, CD11b^+^), neutrophils (Gr-1^+^, CD11c^−^) and natural killer cells (NK1.1^+^) from spleens and bone marrow (BM) of naïve YF77^+/−^, YF16^+/−^, B6.FcγRIIB deficient (RIIB^−/−^) and B6 mice. The results are representative of those from at least two independent experiments.

Figure 2A shows that splenic B cells in these mice express levels of the transgenic FcγRIIB similar to endogenous FcγRIIB levels on B6 splenic B cells, but with somewhat more heterogenous distributions. In the YF77^+/−^ line CD3^+^ splenic T cells also express extremely high levels of the transgenic FcγRIIB, whereas in the YF16^+/−^ line such expression is very low or absent.

Preliminary studies of the T cell dependent (TD) B cell response in the YF77^+/−^ and YF16^+/−^ lines revealed that the primary antibody response in the YF77 line^+/−^ was nearly absent while this response in the YF16^+/−^ line was minimally if at all perturbed as compared to B6 control mice. This result suggested the possibility that the ectopic expression of extremely high levels of the transgenic FcγRIIB on T cells was resulting in gross perturbations of the immune response that were not reflective of normal physiology. As such, we chose not to continue to analyze the YF77^+/−^ line and concentrated our efforts on the YF16^+/−^ line.

Further characterization of the expression of the FcγRIIB Y307→F receptor in the YF16^+/−^ line revealed that it is expressed by splenic and bone marrow (BM) DCs, macrophages and neutrophils, but not by NK cells (Fig. 2B). Expression on the former three cell types is elevated in most cases as compared to the expression of the endogenous receptor on the majority of such cells in B6 mice. Supplemental Figure S1 presents statistical analyses of the flow cytometry data illustrated in Figure 2B.

### Primary B cell development in mice expressing the Y307→F FcγRIIB receptor

**Figure 3:**
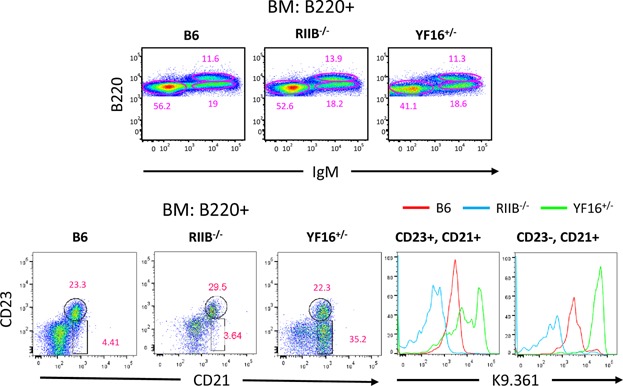
Primary development of B cells in FY16^+/−^ mice in the bone marrow. Panel A: Flow cytometric analysis of total BM B220^+^ cells from mice of the indicated genotypes for surface IgM and B220 levels (upper panels). The percentage of B220^low^ IgM^neg^ pre-pro B, B220^intermediate^ IgM^high^ immature and B220^high^ IgM^low^ mature B cells are shown in three separate gates. The lower panels show the results of analysis of levels of CD23 and CD21/35 on BM B220^+^ cells as well as levels of K9.361 staining on subpopulations expressing different levels of CD23.

FcγRIIB is expressed early in B cell development but there is no known function of this receptor during primary B cell development, and we have not observed major alterations in this development in FcγRIIB deficient mice 31. Examination of stages of primary B cell development in the bone BM of YF16^+/−^ line mice by flow cytometry did not reveal any significant changes in the frequencies of pro, pre and immature B cells (Fig. 3A, upper panels). However, in YF16^+/−^ line mice, a major population of BM B cells expressing low levels of CD23 was observed that was only a minor population in B6 and B6.FcγRIIB deficient mice. Moreover, this subpopulation expressed approximately ten-fold higher levels of the transgenic receptor as compared to expression of the endogenous receptor by B6 BM B cells (Fig. 3A, lower panels). Statistical analyses of the data illustrated in Figure 3A, lower panels are presented in Supplemental Figure S2, middle row.

**Figure 4:**
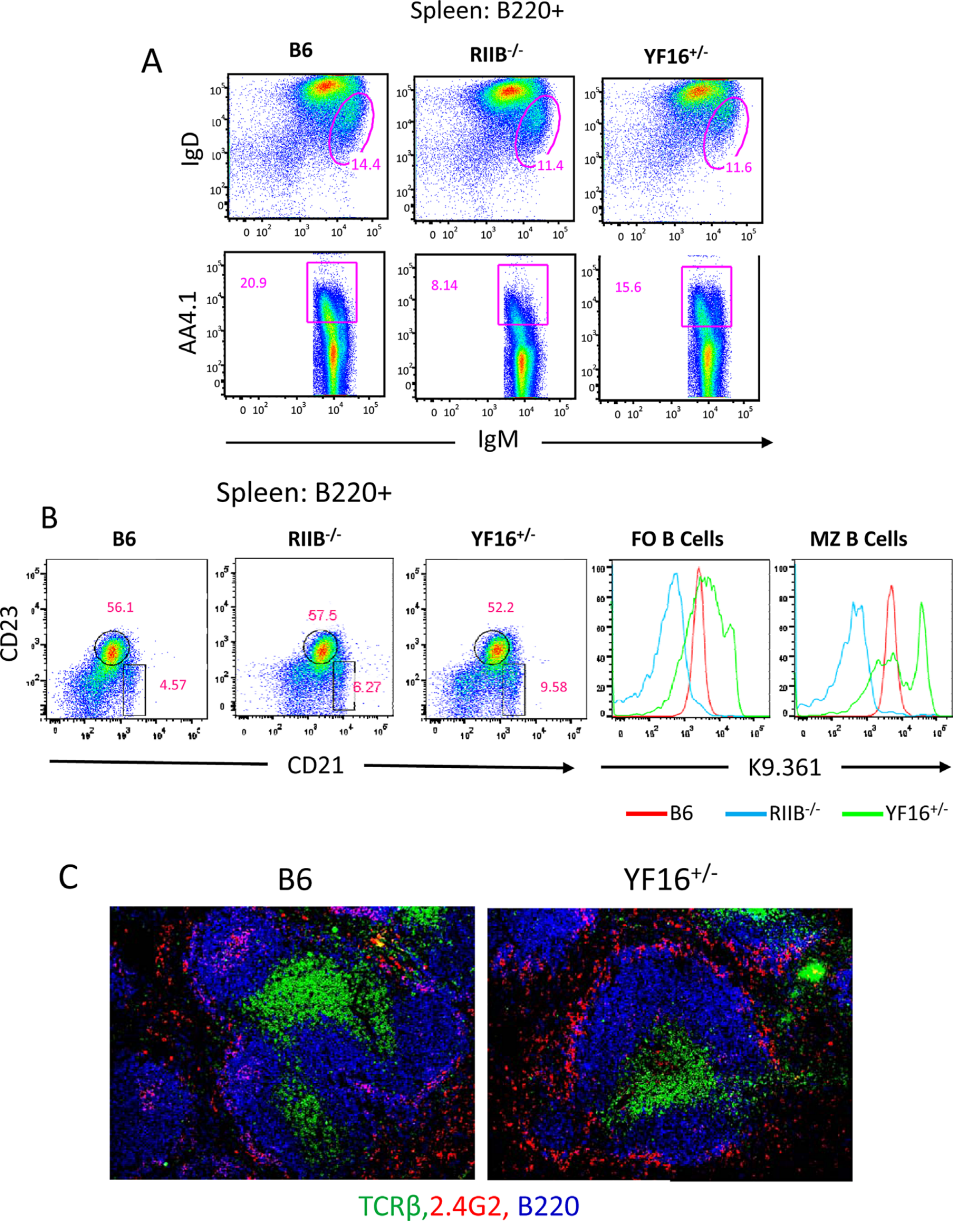
Primary development of B cells in FY16^+/−^ mice in the spleen. A: Flow cytometric analysis of total splenic B220^+^ cells from mice of the indicated genotypes for levels of surface IgD, IgM (upper panels), the transitional marker AA4.1 (anti-C1qRp, lower panels); B: Levels of CD23 and CD21/35 (left panels), and K9.361 (right panels) among cells with a follicular (FO, CD23^high^, CD21^low^) and marginal zone (MZ, CD23^low^, CD21^high^) phenotype; C: Spleen sections obtained from naïve mice of the indicated genotypes were stained with anti-TCR-β (green), 2.4G2 (red), and B220 (blue) and images captured by fluorescence microscopy. Original magnification of images was 200×. All data are representative of those collected from at least two independent experiments.

B cell phenotype and frequency in the spleen of YF16^+/−^ line mice was examined by flow cytometry and histology (Fig. 4). Major differences in B cell phenotype, transitional (AA4.1^+^, Fig. 4A) and mature subset distribution and micro environmental locale were not observed between B6 and YF16^+/−^ line mice. However, a somewhat increased frequency of CD21^high^, CD23^low^ B cells, the phenotype of marginal zone (MZ) B cells, was found in YF16^+/−^ line spleens and a subset of these cells expressed high levels of the mutant FcγRIIB receptor (Fig. 4B). Statistical analyses of the data illustrated in Figure 4B are presented in Supplemental Figure S2, upper row. Histological analyses also revealed an increased frequency of cells expressing high levels of the transgenic FcγRIIB in MZ regions of the spleen of YF16^+/−^ line mice (Fig. 4C). Combined with the analysis of B cells in the BM described above, these data indicate that certain aspects of primary B cell development are somewhat perturbed due to expression of the mutant receptor. However, follicular (FO) B cell development and micro environmental locale appear overtly normal in this line.

**Figure 5:**
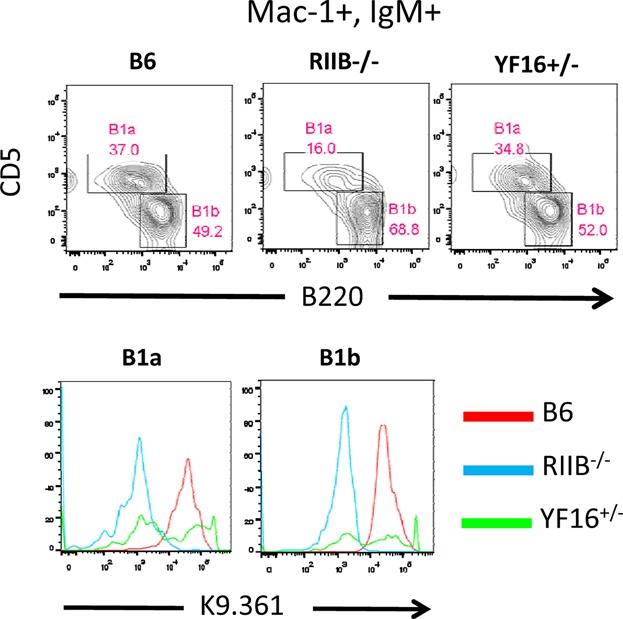
Expression of the Y307→F receptor by peritoneal B cell subpopulations. Peritoneal lavage cells from mice of the indicated genotypes were analyzed for levels of expression of Mac-1, IgM, CD5, B220, and K9.361 staining by flow cytometry. The upper row shows the frequencies of B1a (Mac-1^+^, IgM^+^, B220^+^, CD5^+^) and B1b (Mac-1^+^, IgM^+^, B220^+^, CD5^−^) B1 B cells in mice of the indicated genotypes. The lower row shows levels of K9.361 staining on the B1a and B1b subpopulation in the indicated strains of mice.

As FcγRIIB has been reported to be expressed on peritoneal B1 B cells 46 we also examined expression of the Y307→F transgenic receptor on these cells in YF16^+/−^ mice. Figure 5 shows that YF16^+/−^ mice contain proportions of the two subpopulations of B1 cells (B1a and B1b) that are similar to B6 mice. However, expression of the the Y307→F mutant receptor by these subpopulations is unusual as a major fraction of each appear not to express this receptor, whereas the endogenous receptor is expressed by all such cells in B6 mice. Statistical analyses of the data illustrated in Figure 5, lower row are presented in Supplemental Figure S2, lower row.

### Reduced attenuation of BCR signaling mediated by the Y307→F form of FcγRIIB

As discussed above, co-cross-linking of the BCR and FcγRIIB results in reduction of proximal BCR signaling events, leading to inhibition of downstream signaling and B cell activation. As the Y307→F receptor would be incapable of recruiting SHIP as well as SHP-1 and 2, we expected that co-cross-linking of this receptor to the BCR would not attenuate BCR signaling.

**Figure 6:**
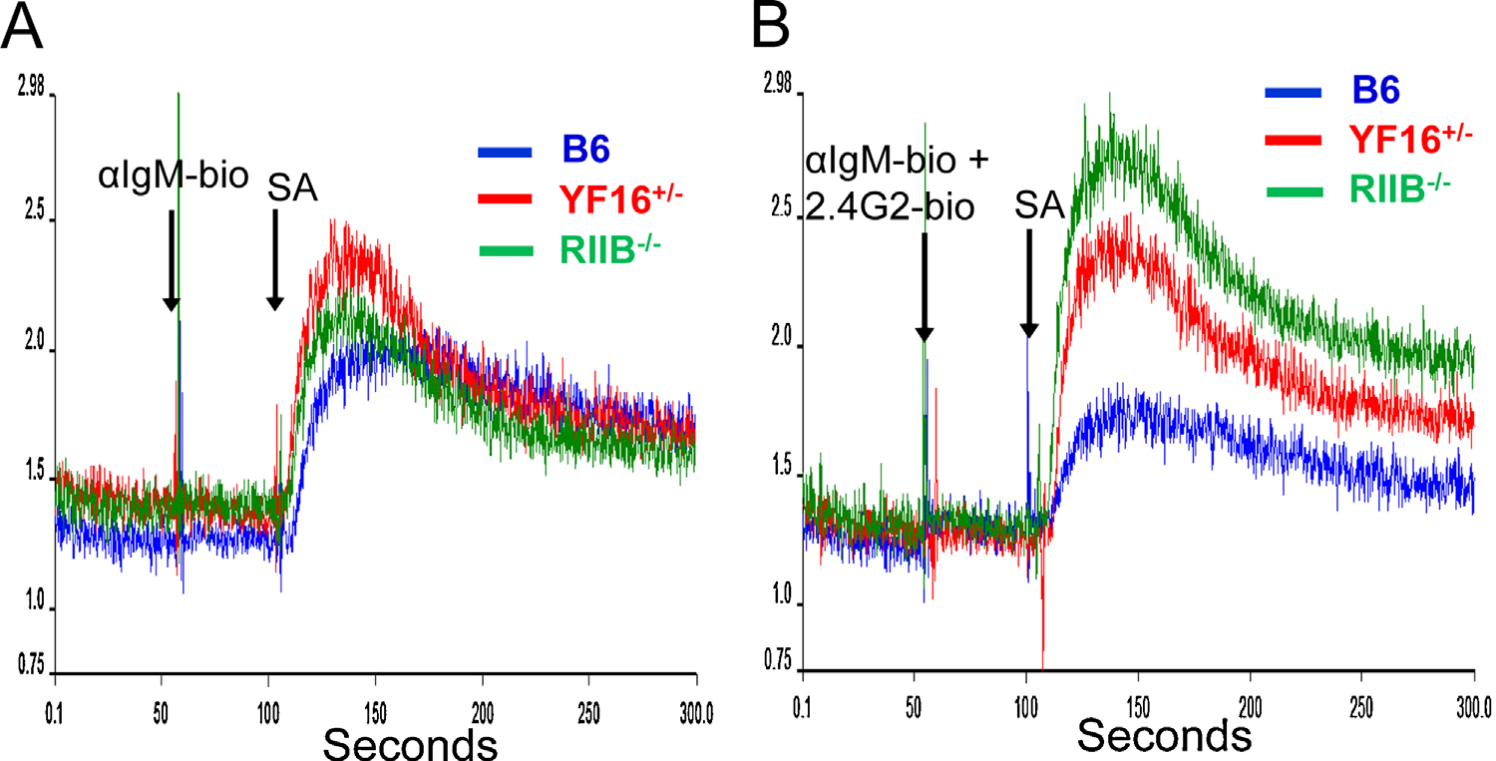
Reduced enhancement of Ca^2+^ flux in YF16^+/−^ B cells after co-cross-linking of the BCR and FcγRIIB as compared to FcγRIIB deficient B cells. Intracellular Ca^2+^ levels were evaluated in purified B cells from mice of the indicated genotypes as described in Materials and Methods. After establishing basal Ca^2+^ levels, either 10 μg/ml of anti-IgM F(ab')_2_ biotin (A) or 10 μg/ml of this reagent and 2.4G2 biotin Abs were added (B), Ca^2+^ levels were continuously monitored, and cross-linking of receptors was achieved by then adding streptavidin to 200 ng/ml followed by continued monitoring. The results are representative of two independent experiments.

To test this idea, splenic B cells were purified from B6, B6.FcγRIIB deficient, and YF16^+/−^ line mice. Intracellular Ca^++^ flux responses of these cells were then monitored after cross-linking of the BCR and co-cross-linking the BCR and FcγRIIB. Figure 6A shows that B cells from these three types of mice fluxed Ca^++^ similarly when the BCR was cross linked, particularly in the persistent stage of this response. Figure 6B shows, as expected, that FcγRIIB deficient B cells displayed substantially elevated BCR-induced Ca^++^ flux as compared to B6 B cells when the BCR and FcγRIIB were co-cross linked. In contrast, YF16^+/−^ line B cells displayed levels of induced Ca^++^ flux intermediate between B6 and B6.FcγRIIB deficient B cells under these conditions, both immediately after co-cross linking and during the persistent stage of the response. The majority of YF16^+/−^ line splenic B cells express surface levels of the Y307→F receptor comparable to or higher than the levels of FcγRIIB expressed by B6 B cells (Figs. 2 and 3), and the extracellular domains of these two receptors are identical. Also, these B cells express similar levels of surface IgM (Fig. 3). As such, these results do not appear to be due to quantitatively reduced levels of BCR-FcγRIIB cross-linking, but to inhibitory functions of the Y307→F receptor that do not depend on phosphorylation of the ITIM motif.

As levels of transgenic FcγRIIB expression is somewhat heterogeneous among splenic B cells (Figs. 4), we FACS purified the predominant population of splenic B cells expression this receptor, which has a follicular B cell surface phenotype, as well as splenic follicular B cells from B6 and FcγRIIB deficient mice and conducted Ca^++^ flux analysis on these cells after co-crosslinking of the BCR and FcγRIIB, as described above. The Ca^++^ flux profiles obtained were similar to those obtained with total splenic B cells (Supplemental Figure S3) suggesting that the contribution of minor subpopulations of B cells expressing highly abnormal levels of the transgenic Y307→F receptor can not account for the intermediate levels of Ca^++^ flux attenuation displayed by the transgenic B cells.

### B cell apoptosis in vitro after homologous cross-linking of wild type and Y307→F mutant forms of FcγRIIB

Previous studies have shown that homologous cross-linking of FcγRIIB on the DT40 B cell line lead to increased levels of apoptosis, and that this outcome is not altered by the Y307→F mutation 25,26. To determine if this was the case for primary B6 and YF16^+/−^ line B cells, purified splenic B cells were either cultured on plates coated with an anti-FcγRIIB mAb, or with a soluble, biotinylated form of this antibody and streptavidin. Levels of apoptosis were then monitored at various time points by flow cytometry. We found that these levels in control cultures were high and were not elevated by homologous cross-linking of either the wild type or mutant forms of FcγRIIB. Moreover, no differences were observed in cultures in which BLyS (BAFF) was added to increase overall levels of B cell survival (data not shown).

### Germinal center responses in FY16^+/−^ line mice

**Figure 7:**
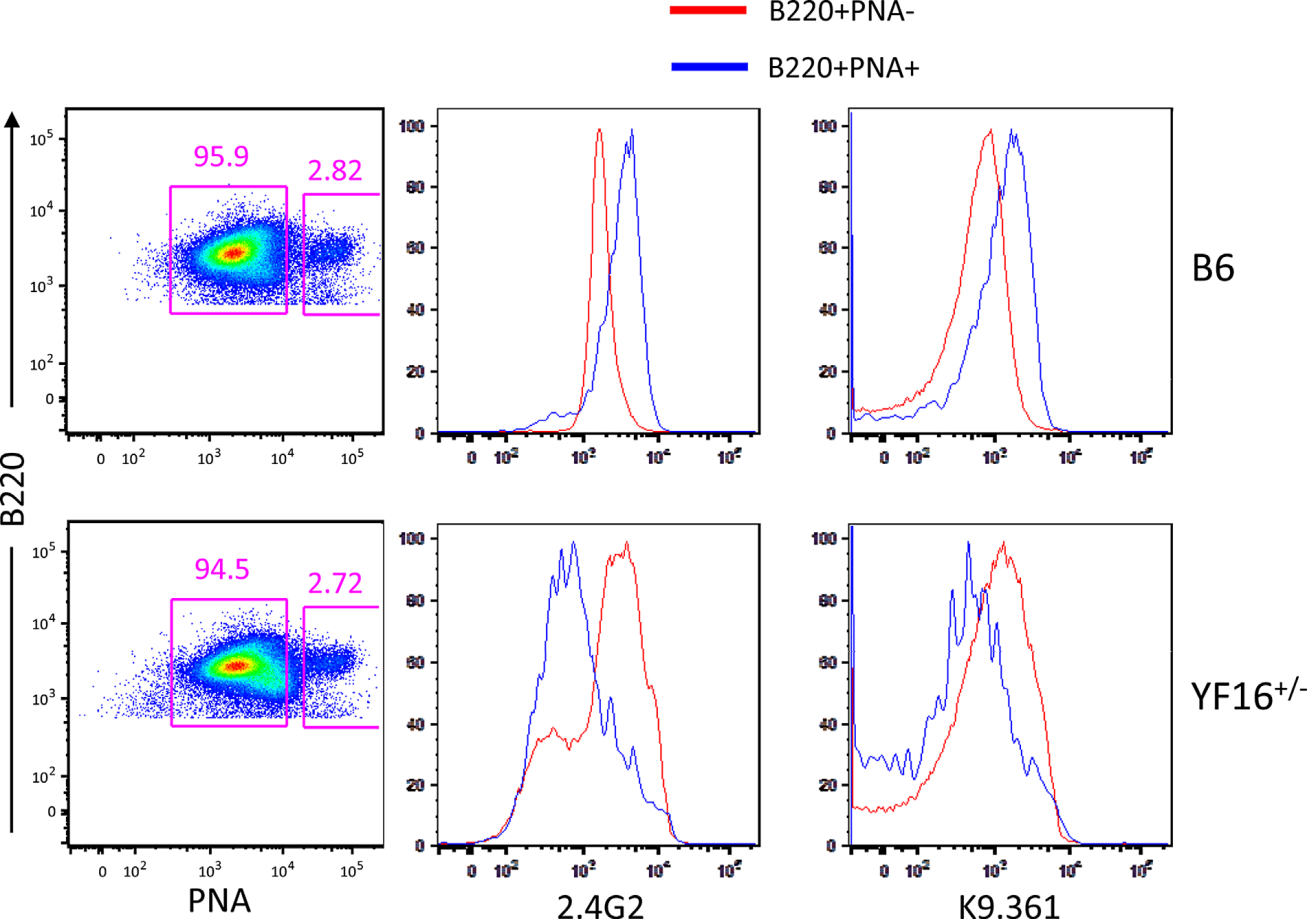
Flow cytometric analysis of FcγRIIB expression on YF16^+/−^ and B6 GC B cells. Splenocytes from SRBC-immunized YF16^+/−^ and B6 mice obtained on day 9 of the immune response were stained with peanut agglutinin (PNA) in combination with anti-B220 and anti-FcγRIIB mAbs 2.4G2 or K9.361. Frequencies of GC (PNA^+^) and non-GC (PNA^−^) B cell populations are shown in the two rectangular gates in the left panels. The levels of staining with 2.4G2 (middle two panels) and K9.361 (right two panels) on non-GC (B220^high^, PNA^−^; red lines) and GC B cells (B220^high^, PNA^+^; blue lines) are shown. These data represent two independent experiments, each including pooled samples from three mice of each strain.

To determine if the germinal center (GC) response was quantitatively altered due to expression of the Y307→F receptor, YF16^+/−^ line mice were immunized with sheep red blood cells (SRBC) and the splenic GC B cell response was evaluated by flow cytometry and histology. We and others have previously shown that FcγRIIB mRNA and surface protein expression are up regulated by GC B cells in B6 mice, but not in several autoimmune prone strains of mice 19,21,47. We also previously showed that the 2.4G2 and K9.361 mAbs do not detectable stain GC B cells in FcγRIIB-deficient mice 21,31. Figure 7 shows that expression of the Y307→F receptor is not up regulated on GC (B220^+^, PNA^+^) B cells in YF16^+/−^ line mice, and in fact may be down modulated, suggesting that the transcriptional control elements necessary for elevated expression are not present in the Y307→F FcγRIIB transgene. This is not surprising, as this transgene contains only FcγRIIB coding sequence, and we and others have obtained data indicating that the “failed” up regulation of FcγRIIB on GC B cells displayed by autoimmune prone strains of mice is due to sequence polymorphisms in non-coding regions of the FcγRIIB gene 19–21,47. Despite these differences in the FcγRIIB ITIM and in FcγRIIB expression by B6 and YF16^+/−^ line GC B cells we did not observe any overt differences in the magnitude of the GC response in these two types of mice.

**Figure 8:**
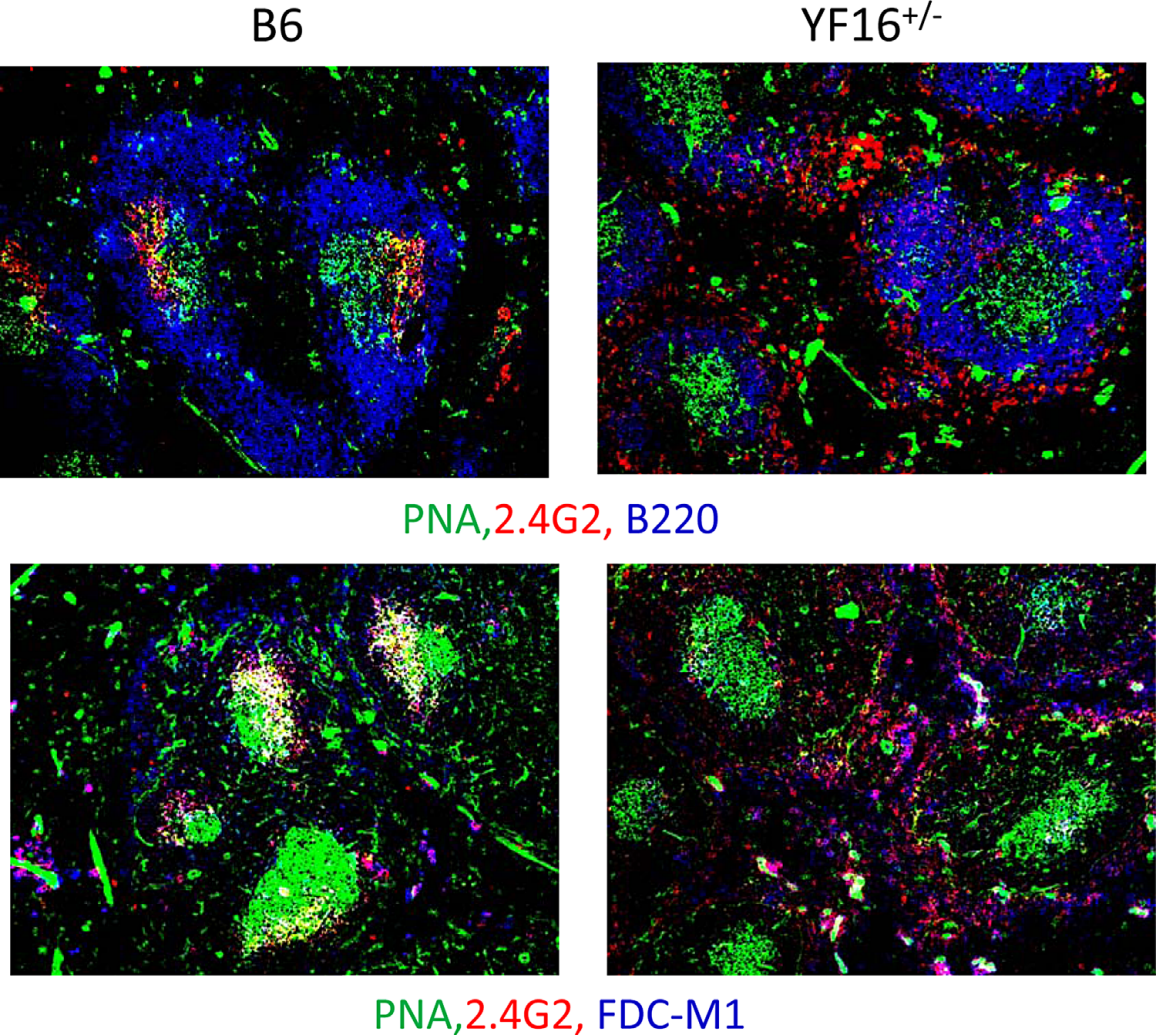
FcγRIIB expression on FDCs in germinal centers of YF16^+/−^ and B6 mice. Spleen sections, obtained from SRBC-immunized YF16^+/−^ and B6 mice on day 9 after immunization, were stained with 2.4G2 (red) and anti-IgD (green) or FDC-M1 (red) and peanut agglutinin (PNA, green). The illustrated images were obtained from GCs in adjacent sections. These data are representative of those obtained from multiple GCs in three mice of each strain. Original magnification of images was 200×. The results are representative of two independent experiments.

The results of histological examination of GCs induced by SRBC immunization of B6 and 16 line mice are shown in Fig. 8. These data revealed that the characteristic up regulation of FcγRIIB expression on FDCs in the so-called light zones of GCs does not take place in YF16^+/−^ line mice. As mentioned above, this failed up regulation is likely due to the absence of the necessary transcriptional control elements in the FcγRIIB transgene. In addition, staining of FDCs with the antibody FDC-M1 is far less intense in the GCs of YF16^+/−^ mice as compared to B6 mice, indicating a possible defect in FDC development or function.

### Y307→F FcγRIIB expression on AFCs in YF16^+/−^ line mice

**Figure 9:**
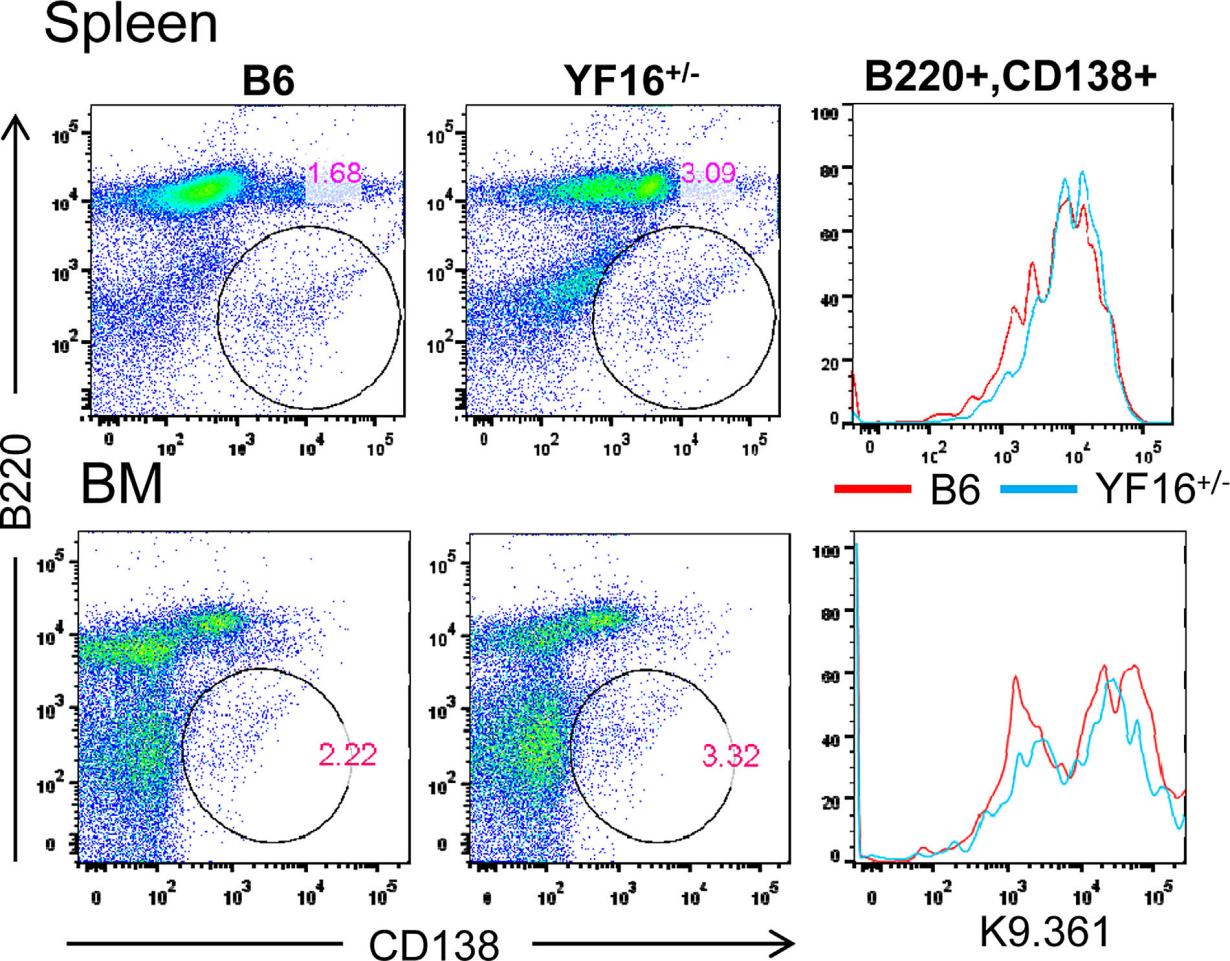
Flow cytometric analysis of FcγRIIB expression on YF16^+/−^ and B6 plasma cells (AFCs). Splenocytes and bone marrow cells from SRBC-immunized YF16^+/−^ and B6 mice on day 9 of the immune response were stained with anti-CD138 (syndecan-1) in combination with anti-B220 and K9.361. AFCs were defined as B220^low^ CD138^+^ (spleen; upper left panels, and bone marrow; lower left panels). The levels of staining with K9.361 (right two panels) on B6 AFCs and YF16^+/−^ AFCs are shown. These data represent two independent experiments, each including pooled samples from three mice of each strain.

While some studies have reported that an FcγRIIB deficiency in mice results in quantitative and qualitative changes in the GC response 48,49 we have not observed such differences 31. In contrast, past studies are in agreement that FcγRIIB deficiencies result in amplification of the AFC and serum antibody responses, particularly the IgG response, to foreign as well as auto antigens 4,17,22,27,28,34,40,41. To investigate the influence of expression of the Y307→F form of FcγRIIB on the AFC response we first determined whether this mutant receptor was present on AFCs in the YF16^+/−^ line. Figure 9 shows that this receptor is expressed on AFCs induced in vivo by immunization of YF16^+/−^ line mice with SRBCs at levels comparable to those on AFCs in B6 mice. We also found that AFCs induced in vitro by cultivation of purified YF16^+/−^ line and B6 splenic B cells with LPS and IL-4 also express this receptor at similar levels (data not shown).

### Amplified long lived AFC and anamnestic AFC responses in FY16^+/−^ line mice

To evaluate the influence of the Y307→F FcγRIIB receptor on the AFC response in vivo, YF16^+/−^ line mice were immunized with NP-CGG and splenic and BM AFC responses and serum antibody levels evaluated 2 weeks later as compared to B6 and B6.FcγRIIB deficient mice. Groups of mice were also rested for 4 weeks after priming with NP-CGG. Anti-NP AFC and serum antibody responses were then assayed in half of these rested mice (long-lived AFCs and antibody). The other half of the cohort was boosted with NP-CGG in saline and AFC responses and serum antibody levels assayed 7 days later (memory AFCs and antibody).

**Figure 10:**
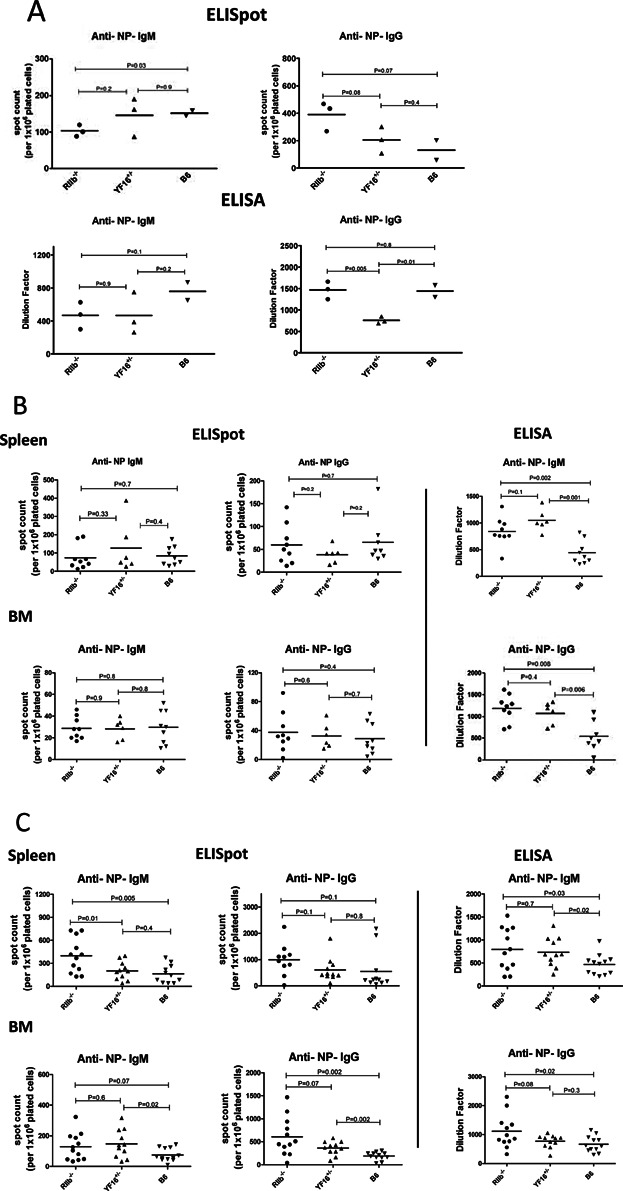
AFC and serum Ab responses in FY16^+/−^ mice. B6.FcγRIIB deficient (RIIB^−/−^), YF16^+/−^ and B6 mice were immunized with NP-CGG as described in Materials and Methods. A: The primary anti-NP IgM and IgG antibody response was measured by ELISA on day 14 post immunization as well as the number of NP specific IgM (upper left panel) and IgG (upper right panel) secreting AFCs measured by ELISpot assay as described in Materials and Methods. Each point in the ELISpot results represents the number of AFCs per 1 × 10^6^ splenocytes obtained from an individual mouse. Each point in the ELISA results represents the dilution factor giving an OD value of 1.0 (450 nm) obtained from the serum of a single mouse. B: Long lived (day 30 post immunization), and; C: Memory (day 7 after boost) AFC responses in spleen and bone marrow and NP specific total Igs in serum were quantified and illustrated as described above. Statistical analysis was performed by Student's *t*-test. The data for long-lived and memory AFCs were obtained from three independent experiments.

No clear pattern of differences in the primary anti-NP IgM and IgG splenic AFC and serum antibody responses were observed among FcγRIIB deficient, B6, and YF16^+/−^ line mice (Fig. 10A). In contrast, long lived serum IgM and IgG antibody levels were elevated in YF16^+/−^ line mice to an extent similar to that observed in B6.FcγRIIB deficient mice – approximately twofold (Fig. 10B, right panels). However, these differences were not reflected by variations in the levels of the respective types of AFCs as measured by ELISpot assay (Fig. 10B, left panels).

After boosting, IgM and IgG splenic anti-NP AFC levels were higher in B6.FcγRIIB deficient mice than in B6 mice but this was not the case in YF16^+/−^ line mice (Fig. 10C). However, IgM and IgG BM anti-NP AFC levels were elevated in both B6.FcγRIIB deficient and YF16+/− line mice as compared to B6 mice, although those levels in YF16^+/−^ line mice appeared less than in B6.FcγRIIB deficient mice. This trend was also observed among IgM and IgG serum antibodies in the various strains, where B6.FcγRIIB deficient mice had the highest levels and B6 mice the lowest levels, with sera from YF16^+/−^ line mice appearing to contain roughly intermediate levels of these antibodies. In total, these data indicate that expression of the Y307→F mutant FcγRIIB receptor results in elevated antibody responses, but not to the same extent as from a complete FcγRIIB deficiency. In all of the ELISA assays an NP-BSA conjugate capture reagent containing on average of 19 NP haptens per BSA molecule was used. As this should allow even low affinity serum anti-NP antibodies to be detected in the assay it is unlikely that the differences we observed in the ELISA assays can be accounted for by differences in the average affinities of anti-NP antibody in the various sera.

### Antinuclear antibody production in aged YF16^+/−^ mice

Previous studies have shown that a deficiency in FcγRIIB contributes to the development of serum antinuclear antibodies (ANAs) in autoimmune-prone strains of mice 34,50. On the pure B6 (nonautoimmune) background, however, the penetrance of this effect is rather weak, with a few B6.FcγRIIB deficient mice developing significant ANA titers at advanced age 51. To determine if the Y307→F form of FcγRIIB alters this penetrance, three B6.FcγRIIB deficient and three YF16^+/−^ mice were aged, bled at 18, 32, and 54 weeks of age, and serum assayed for the presence of ANA antibodies. In each group of mice only one developed detectable ANA titers at 54 weeks of age. While larger numbers of mice would need to be analyzed for definitive results to be obtained, these data indicate that expression of the Y307→F transgenic form of FcγRIIB does not dramatically alter the influence of an FcγRIIB deficiency on ANA production.

## DISCUSSION

To test the role of the FcγRIIB ITIM motif in vivo, we created lines of mice expressing transgenes encoding a mutant form of FcγRIIB1 with an inactivating Y to F mutation in this motif. Two lines were obtained using different transgenic constructs that displayed transgenic FcγRIIB expression on most B cells that was comparable to expression levels of endogenous FcγRIIB on B cells in B6 mice. However, both lines of mice had abnormal expression of the transgenic receptor in other lineages. In the YF77^+/−^ line, T cells, that lack expression of FcγRIIB in normal mice, had high-level expression of the mutant transgenic FcγRIIB. Antibody responses in YF77^+/−^ line mice were dramatically reduced, indicating gross perturbations of immune function. As such, we reasoned that further analysis of this line would not provide useful information regarding the normal *in vivo* function of the FcγRIIB ITIM motif.

In the YF16^+/−^ line, in contrast, we did not detect ectopic expression of the mutant FcγRIIB receptor on T cells but this receptor was expressed at elevated levels on several hematopoietic cell types that normally express the endogenous receptor, and expression of the transgenic receptor was not detectable on FDCs in GCs. We, and others have previously shown that FDCs are induced to express very high levels of endogenous FcγRIIB during the GC reaction 17,18. Moreover, the expression of the transgenic FcγRIIB receptor was not up regulated on GC B cells, as we and others have shown is the case for the endogenous receptor in autoimmune strains of mice 20,21,47. We also observed that a subset of splenic MZ B cells expressed elevated levels of the transgenic receptor and the majority of an expanded population BM B cells with a CD23^low^ phenotype also did so. The potential impact of these alterations on TD immune responses is difficult to predict. However, FO B cells usually predominate the response to TD antigens such as SRBC and NP-CGG, and this subpopulation appeared overtly normal in phenotype and frequency in YF16^+/−^ line mice. Nonetheless, we must consider that some of the differences we observed in B cell immune responses in the YF16^+/−^ mice as compared to controls are due to the abnormal expression levels of the transgenic FcγRIIB receptor on either B cells, accessory cells, or both.

We detected no quantitative alterations of the GC response in the YF16^+/−^ line. This result is in keeping with our previous findings that lack of expression of the endogenous FcγRIIB receptor on B cells does not quantitatively alter the GC response 31. We also previously found no effect of lack of B cell expression of the endogenous FcγRIIB receptor on negative selection during the GC reaction of a B cell clone expressing an autoreactive BCR 31. In contrast, data from other laboratories have implicated FcγRIIB in the action of peripheral B cell tolerance checkpoints operative in the GC 48,49. Further studies will be required to resolve these discrepancies and to rigorously test a possible role for the FcγRIIB ITIM motif in regulation of the GC reaction. However, one of the predictions of previous in vitro studies of FcγRIIB activity is that inactivation of the ITIM motif could result in unbridled activity of the apoptosis inducing function of FcγRIIB 25. This might have been manifested in a quantitatively reduced GC B cell response but this was not observed in the YF16^+/−^ line. Also, we did not detect an increased level of apoptosis in purified YF16^+/−^ line B cells when the Y307→F mutant FcγRIIB receptor was extensively cross-linked in vitro. As such, whether this receptor can induce apoptosis at all stages of B cell differentiation in vivo requires more detailed examination. In this regard, the apoptosis inducing activity of FcγRIIB has been well described in the transformed chicken B cell line DT40 in vitro 25,26, but reported levels of apoptosis resulting from homologous cross-linking of this receptor on mouse splenic primary B cells, AFCs induced in vitro or cultured ex vivo*,* and purified B1a B cells have been rather low 25,27,46.

The conclusions of numerous previous studies, including our own, agree that a primary role for FcγRIIB is regulation of the magnitude and persistence of the antibody response produced by AFCs 4,27–31. This finding was originally made with FcγRIIB deficient lines of mice produced using ES cells generated from strain 129 mice 28,29. Subsequently, it was discovered that 129 allelic forms of genes tightly linked to the endogenous FcγRIIB locus (i.e., those in the Sle16 region) also altered regulation of serum autoantibody antibody levels 52. Generation of new lines of FcγRIIB deficient mice using B6 ES cells has helped to resolve the relative roles of the 129 linked alleles and FcγRIIB in the regulation of serum antibody levels 51. These mice display elevated spontaneous autoantibody serum antibody levels but these do not approach the levels observed in mice also containing the 129 Sle16 region. Our results, obtained using an FcγRIIB deficient line constructed using B6 ES cells, are in keeping with these findings. We observed moderate twofold increases in the induced long term and memory serum and AFC response IgM and IgG in these mice (Fig. 10). We also found that some B6.FcγRIIB deficient mice developed significant serum antinuclear antibody levels and only at advanced age.

The FY16^+/−^ line was constructed on a B6 background, and then crossed to the FcγRIIB deficient line created using B6 ES cells. As such, differences in alleles in the Sle16 region would not have influenced the results we obtained in these studies. We found that both splenic and BM AFCs in the FY16^+/−^ line express levels of the mutant FcγRIIB receptor comparable to expression of the endogenous receptor in these types of AFCs in B6 mice (Fig. 9). Despite this, differences in the long lived and memory serum antibody and AFC responses were observed among YF16^+/−^ line and B6 and B6.FcγRIIB deficient mice, suggesting that the absence of a functional ITIM motif in FcγRIIB contributed to these differences. Six weeks after immunization with NP-CGG, serum anti-NP IgM and IgG in both B6.FcγRIB deficient and YF16^+/−^ line mice were similarly twofold elevated as compared to B6 mice, indicating that a functional ITIM motif is required for the regulation of persistent serum antibody levels. However, mechanistic interpretation of this finding is complicated by the fact that we observed no differences in the frequency of IgM or IgG anti-NP AFCs 6 weeks after immunization among any of the strains. It is possible that the amount of antibody produced per AFC may be altered by absence of FcγRIIB or expression of the mutant receptor, or that the AFCs responsible for the elevated serum antibody levels are resident in tissues other than the spleen and BM.

More complex results were obtained from mice primed with NP-CGG, boosted at 4 weeks, and then analyzed 1 week later. In this case, B6.FcγRIIB deficient mice showed approximately two-fold increases in both IgM and IgG spleen and BM anti-NP AFCs and serum antibody levels as compared to B6 mice, but YF16^+/−^ line mice displayed an intermediate phenotype, with anti-NP AFC and serum antibody levels that were moderately increased compared to B6 mice in some assays but not others.

Finally, in a small scale experiment the frequency of mice that developed spontaneous serum ANA titers at 8 months of age did not differ between B6.FcγRIIB deficient mice and YF16^+/−^ mice. This indicates that expression of the Y307→F mutant form of FcγRIIB is not sufficient to suppress the autoantibody production resulting from an FcγRIIB deficiency.

In total, our data suggest that the ITIM Y307→F mutant form of FcγRIIB retains some inhibitory regulatory activity for foreign antigen induced AFC responses in vivo. This is in keeping with our in vitro results showing that BCR signaling in YF16^+/−^ line B cells is not amplified to the same extent as observed for FcγRIIB deficient B cells, when the Y307→F mutant receptor is co-cross-linked to the BCR. This residual inhibition might be accounted for by the apoptosis inducing activity of FcγRIIB, or its ability to inhibit formation of the immunological synapse on B cells. With regard to the former possibility, however, as mentioned above, both our in vitro and in vivo experiments failed to provide evidence for elevated levels of cell death among activated B cells expressing the Y307→F receptor. Nonetheless, further studies to more rigorously test this idea are clearly warranted. Finally, confounding influences of the abnormal levels of expression of the mutant receptor on some types of hematopoietic cells and failure of its levels of expression to be regulated in B cells and FDCs as is the endogenous receptor must also be considered. For example, lack of expression of the Y307→F mutant receptor on FDCs in GCs could perturb antigen-containing IC trapping and retention in this microenvironment, or other aspects of FDC function, perhaps influencing the tempo and extent of commitment of GC B cells to the AFC pathway 53. Resolving these issues will require the generation of mice in which tissue distribution and levels of expression of an ITIM Y307→F mutant FcγRIIB are regulated as is the endogenous receptor.

## Supporting Information

Figure S1 : Mean fluorescence intensity of K9.361staining (anti-B6 FcγRIIB) on various cell types. The Mean Fluorescence intensity (MFI) of K9.361 (anti-B6 FcγRIIB) staining were determined on dendritic cells (CD11b/11c^+^), macrophages (F4/80^+^, CD11b^+^), neutrophils (Gr-1^+^, CD11c^−^) and natural killer cells (NK1.1^+^) from spleens and bone marrow (BM) of naïve YF77^+/−^, YF16^+/−^, B6.FcγRIIB deficient (RIIB^−/−^) and B6 mice. The graphs are representative of the mean and standard deviation (SD) of MFI from each group of mice (n = 3). Statistics were calculated using paired *t*-test.

Figure S2 : Mean fluorescence intensity of K9.361 (anti-B6 FcγRIIB) staining on various cell types in various organs. The Mean Fluorescence intensity (MFI) of K9.361 (anti-B6 FcγRIIB) staining was determined on: BM follicular (FO, CD23^high^, CD21^low^) and marginal zone (MZ, CD23^low^, CD21^high^) phenotype (upper panel); Spleen follicular (FO, CD23^high^, CD21^low^) and marginal zone (MZ, CD23^low^, CD21^high^) phenotype (middle panel); peritoneal B1a (Mac-1^+^, IgM+, B220^+^, CD5^+^) and B1b (Mac-1^+^, IgM^+^, B220^+^, CD5^−^) B cells (lower panel) from naïve YF77^+/−^, YF16^+/−^, B6.FcγRIIB deficient (RIIB^−/−^) and B6 mice. The graphs are representative of the mean and standard deviation (SD) of MFI from each group of mice (n = 3). Statistics were calculated using paired *t*-test.

Figure S3 : Reduced enhancement of Ca^2+^ flux in follicular B cells of YF16^+/−^ mice after co-cross-linking of the BCR and FcγRIIB. Follicular splenic B cells (FO) were FACS purified using B220^+^, CD23^high^, CD21^low^ gating. Intracellular Ca^2+^ levels were evaluated in sorted FO B cells from mice of the indicated genotypes as described in Materials and Methods. After establishing basal Ca^2+^ levels, 10 μg/ml of both anti-IgM (Fab) biotin and 2.4G2 biotin Abs were added, and cross-linking of receptors was achieved by then adding streptavidin to 200 ng/ml followed by continued monitoring of Ca^2+^ levels.
