# Supplementary figures and images for: Testing the role of the FcγRIIB immunoreceptor tyrosine-based inhibitory motif in regulation of the B cell immune response
Source: Immun Inflamm Dis. 2015 Jun 4;3(3):247–64. doi: 10.1002/iid3.64 (PMC4578524; doi:10.1002/iid3.64)

## Slide 1
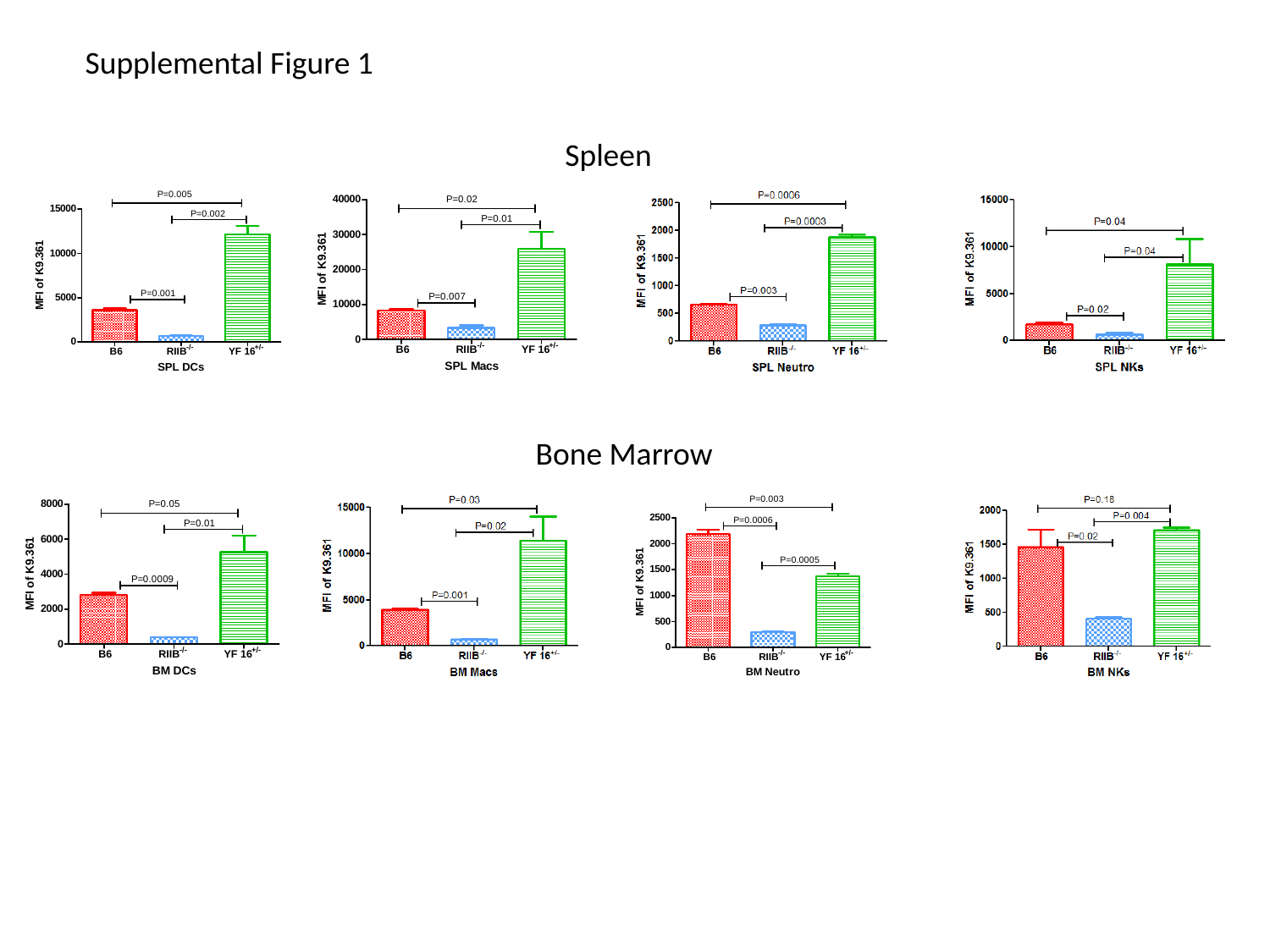

Supplemental Figure 1
Spleen
Bone Marrow

Supplement: Figure S1 — : Mean fluorescence intensity of K9.361staining (anti-B6 FcγRIIB) on various cell types. The Mean Fluorescence intensity (MFI) of K9.361 (anti-B6 FcγRIIB) staining were determined on dendritic cells (CD11b/11c+), macrophages (F4/80+, CD11b+), neutrophils (Gr-1+, CD11c−) and natural killer cells (NK1.1+) from spleens and bone marrow (BM) of naïve YF77+/−, YF16+/−, B6.FcγRIIB deficient (RIIB−/−) and B6 mice. The graphs are representative of the mean and standard deviation (SD) of MFI from each group of mice (n = 3). Statistics were calculated using paired t-test. [file iid30003-0247-sd1.pptx]

## Slide 1
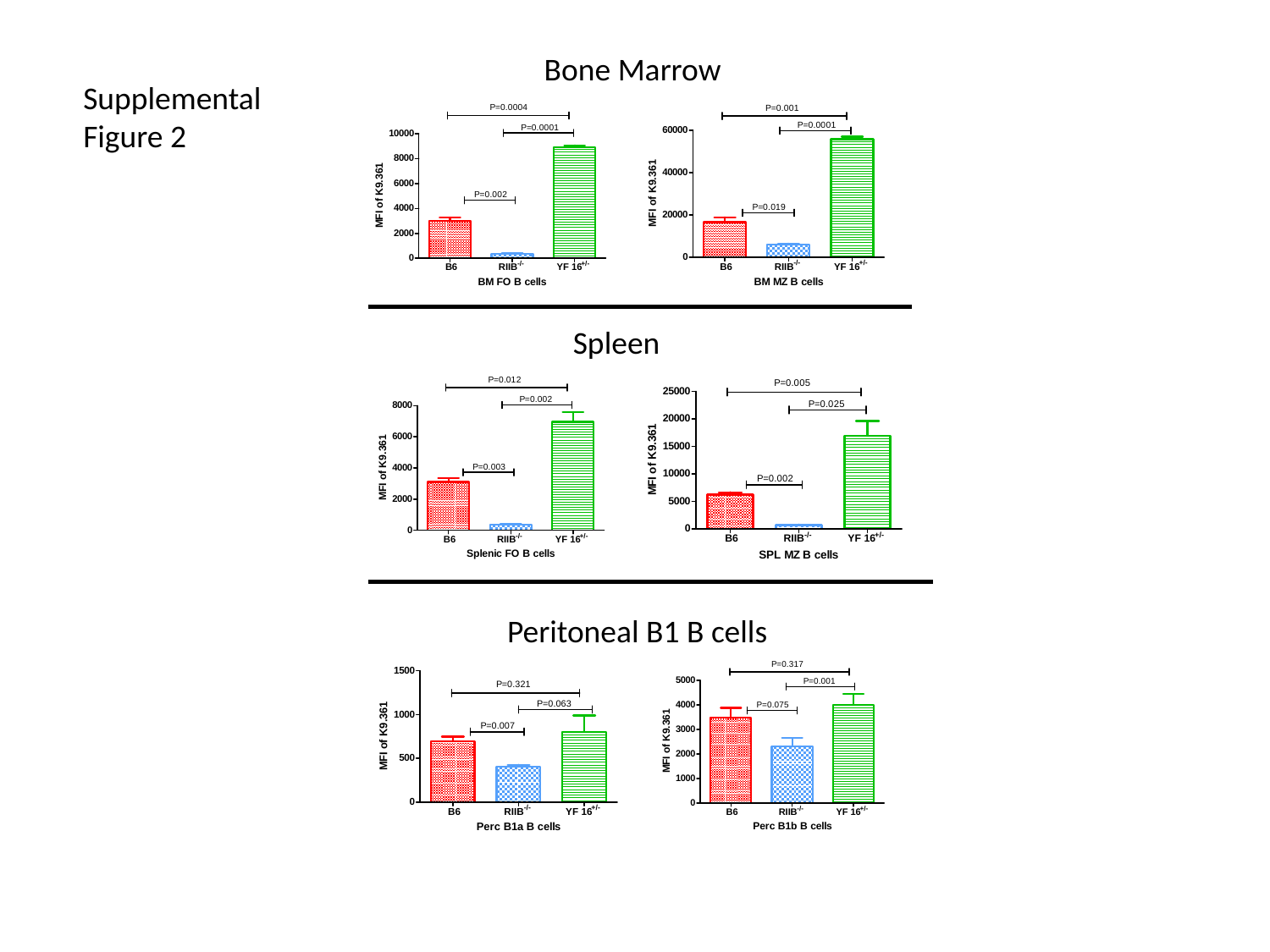

Bone Marrow
Supplemental
Figure 2
Spleen
Peritoneal B1 B cells

Supplement: Figure S2 — : Mean fluorescence intensity of K9.361 (anti-B6 FcγRIIB) staining on various cell types in various organs. The Mean Fluorescence intensity (MFI) of K9.361 (anti-B6 FcγRIIB) staining was determined on: BM follicular (FO, CD23high, CD21low) and marginal zone (MZ, CD23low, CD21high) phenotype (upper panel); Spleen follicular (FO, CD23high, CD21low) and marginal zone (MZ, CD23low, CD21high) phenotype (middle panel); peritoneal B1a (Mac-1+, IgM+, B220+, CD5+) and B1b (Mac-1+, IgM+, B220+, CD5−) B cells (lower panel) from naïve YF77+/−, YF16+/−, B6.FcγRIIB deficient (RIIB−/−) and B6 mice. The graphs are representative of the mean and standard deviation (SD) of MFI from each group of mice (n = 3). Statistics were calculated using paired t-test. [file iid30003-0247-sd2.pptx]

## Slide 1
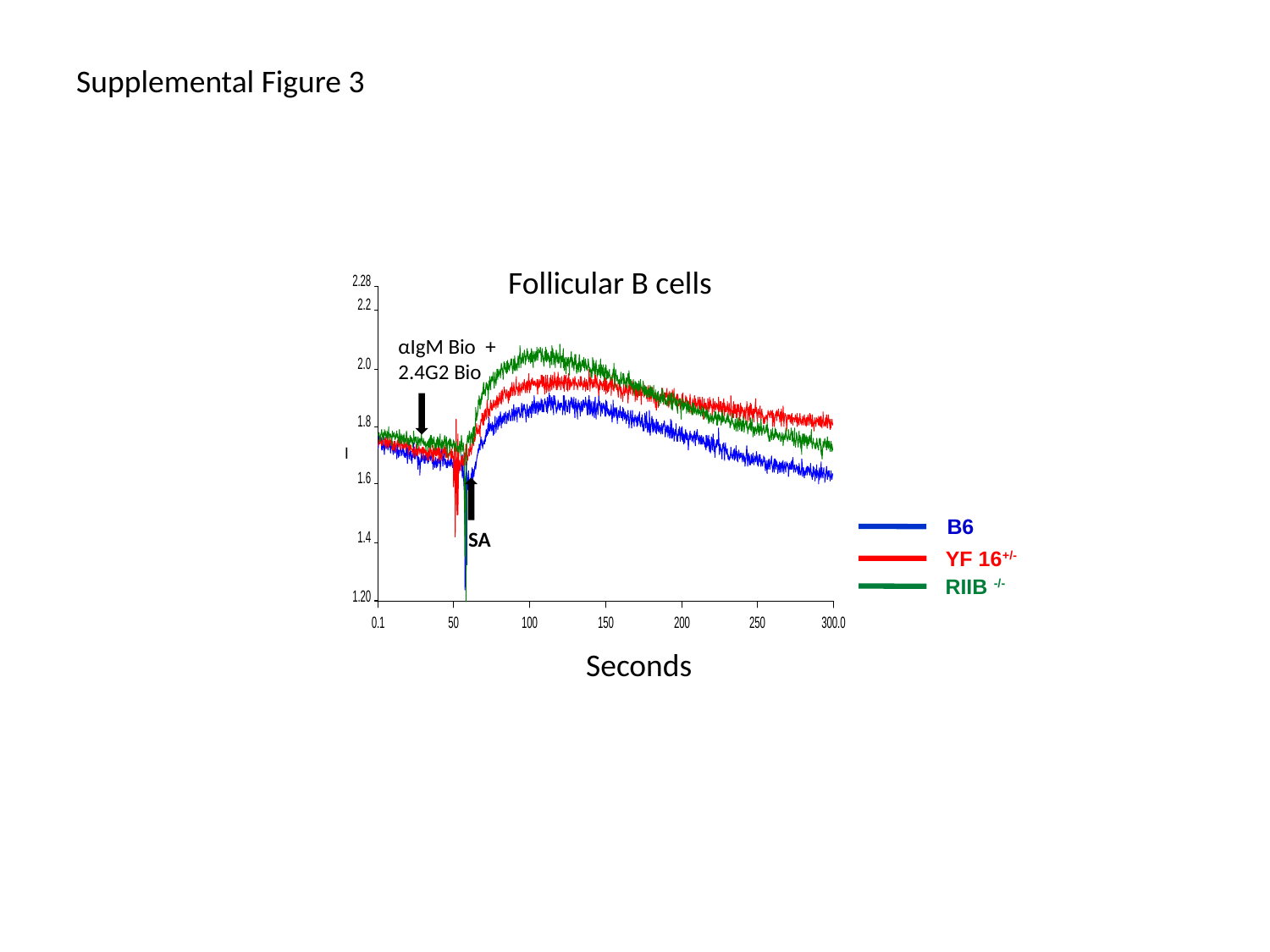

Supplemental Figure 3
Follicular B cells
αIgM Bio +
2.4G2 Bio
SA
Seconds
B6
YF 16+/-
RIIB -/-

Supplement: Figure S3 — : Reduced enhancement of Ca2+ flux in follicular B cells of YF16+/− mice after co-cross-linking of the BCR and FcγRIIB. Follicular splenic B cells (FO) were FACS purified using B220+, CD23high, CD21low gating. Intracellular Ca2+ levels were evaluated in sorted FO B cells from mice of the indicated genotypes as described in Materials and Methods. After establishing basal Ca2+ levels, 10 μg/ml of both anti-IgM (Fab) biotin and 2.4G2 biotin Abs were added, and cross-linking of receptors was achieved by then adding streptavidin to 200 ng/ml followed by continued monitoring of Ca2+ levels. [file iid30003-0247-sd3.pptx]
